# HIF-1-Dependent Induction of β3 Adrenoceptor: Evidence from the Mouse Retina

**DOI:** 10.3390/cells11081271

**Published:** 2022-04-08

**Authors:** Rosario Amato, Francesco Pisani, Emiliano Laudadio, Maurizio Cammalleri, Martina Lucchesi, Silvia Marracci, Luca Filippi, Roberta Galeazzi, Maria Svelto, Massimo Dal Monte, Paola Bagnoli

**Affiliations:** 1 Department of Biology, University of Pisa, 56127 Pisa, Italy; rosario.amato@biologia.unipi.it (R.A.); maurizio.cammalleri@unipi.it (M.C.); martina.lucchesi@student.unisi.it (M.L.); silvia.marracci@unipi.it (S.M.); paola.bagnoli@unipi.it (P.B.); 2 Department of Biosciences, Biotechnologies and Biopharmaceutics, University of Bari Aldo Moro, 70125 Bari, Italy; francesco.pisani@uniba.it (F.P.); maria.svelto@uniba.it (M.S.); 3 Department of Materials, Environmental Sciences and Urban Planning, Polytechnic University of Marche, 60131 Ancona, Italy; e.laudadio@staff.univpm.it; 4 Department of Clinical and Experimental Medicine, Division of Neonatology and NICU, University of Pisa, 56100 Pisa, Italy; luca.filippi@unipi.it; 5 Department of Life and Environmental Sciences, Polytechnic University of Marche, 60131 Ancona, Italy; r.galeazzi@staff.univpm.it; 6 Institute of Biomembranes and Bioenergetics, National Research Council, 70126 Bari, Italy; 7 National Institute of Biostructures and Biosystems (INBB), 00136 Rome, Italy

**Keywords:** oxygen-induced retinopathy, HIF-1 binding site, computational analysis, ChIP-qPCR, gene expression

## Abstract

A major player in the homeostatic response to hypoxia is the hypoxia-inducible factor (HIF)-1 that transactivates a number of genes involved in neovessel proliferation in response to low oxygen tension. In the retina, hypoxia overstimulates β-adrenoceptors (β-ARs) which play a key role in the formation of pathogenic blood vessels. Among β-ARs, β3-AR expression is increased in proliferating vessels in concomitance with increased levels of HIF-1α and vascular endothelial growth factor (VEGF). Whether, similarly to VEGF, hypoxia-induced β3-AR upregulation is driven by HIF-1 is still unknown. We used the mouse model of oxygen-induced retinopathy (OIR), an acknowledged model of retinal angiogenesis, to verify the hypothesis of β3-AR transcriptional regulation by HIF-1. Investigation of β3-AR regulation over OIR progression revealed that the expression profile of β3-AR depends on oxygen tension, similar to VEGF. The additional evidence that HIF-1α stabilization decouples β3-AR expression from oxygen levels further indicates that HIF-1 regulates the expression of the β3-AR gene in the retina. Bioinformatics predicted the presence of six HIF-1 binding sites (HBS #1-6) upstream and inside the mouse β3-AR gene. Among these, HBS #1 has been identified as the most suitable HBS for HIF-1 binding. Chromatin immunoprecipitation-qPCR demonstrated an effective binding of HIF-1 to HBS #1 indicating the existence of a physical interaction between HIF-1 and the β3-AR gene. The additional finding that β3-AR gene expression is concomitantly activated indicates the possibility that HIF-1 transactivates the β3-AR gene. Our results are indicative of β3-AR involvement in HIF-1-mediated response to hypoxia.

## 1. Introduction

Oxygen sensors play a primary role in living organisms as they activate homeostatic responses when oxygen levels decrease. Oxygen availability is sensed by oxygen-sensitive enzymes, which, in turn, regulate the activity of transcription factors. Among these, the first to be identified was hypoxia-inducible factor (HIF)-1 [1]. HIF-1 is a dimer formed by a labile α and a stable β subunits. In the presence of oxygen, HIF-1α is hydroxylated by oxygen-sensitive prolyl hydroxylases, ubiquitinated by the von Hippen–Lindau protein and therefore degraded by the proteasome. When oxygen levels drop, HIF-1α escapes from hydroxylation and stabilizes, migrates into the nucleus, dimerizes with HIF-1β and binds to the hypoxia-responsive elements (HREs). HREs are composite regulatory elements containing conserved HIF-binding sites (HBSs) and highly variable flanking sequences [2]. HIF-1 promotes the transactivation of hundreds of genes involved in metabolic adaptation to reduced oxygen availability [3]. One of the main HIF-1 target genes is that of VEGF, which plays a major role in triggering angiogenesis as metabolic adaptation to hypoxia consisting in the local formation of new blood vessels from pre-existing ones [4]. Oxygen-dependent angiogenesis is particularly relevant in tissues characterized by high rate of oxygen consumption, as the case of the retina. In effect, hypoxia-driven angiogenesis is crucial for retinal vascularization during development but is also implied in the pathogenesis of several retinal diseases such as proliferative retinopathies [5]. The hypoxia-driven angiogenesis is influenced by several side mechanisms, which could enhance or inhibit the activity of the HIF-1/VEGF axis.

At the retinal level, β-adrenoceptors (β-ARs) have been established to cover a relevant role in the regulation of hypoxia-driven angiogenesis [6]. In particular, hypoxia determines the surge of norepinephrine which drives the overstimulation of β-ARs [7]. Among β-ARs, particular attention has been recently directed to β3-AR, the third β-AR subtype to be discovered that was cloned and characterized in humans at the end of the eighties [8]. β3-AR’s role in response to hypoxia has been determined in several tissues supporting the hypothesis that reduced oxygen tension may trigger β3-AR upregulation in hypoxic conditions. In particular, β3-AR is highly expressed in embryo tissues, which are physiologically hypoxic [9], while showing a restricted expression in adult tissues [10] in which β3-AR levels increase in conditions associated with hypoperfusion, such as cancer or heart failure [11,12,13]. In this respect, there is evidence that hypoxia increases β3-AR expression in endothelial cells lining proliferating vessels, as for instance in the human heart or in the mouse retina. In heart failure in particular, β3-AR is upregulated in the hypoxic endothelium of coronary arteries where it is involved in vasodilation and re-vascularization through the nitric oxide pathway [14]. In the hypoxic retina, β3-AR is localized to proliferating vessels where its expression is drastically upregulated when oxygen tension decreases [15]. Assuming that β3-AR expression is regulated by oxygen tension, then the possibility exists that β3-AR upregulation in response to hypoxia involves HIF-1-mediated transcriptional mechanisms as it occurs for additional hypoxia-inducible proteins.

In the present study, the hypothesis that HIF-1 may act as a transcription factor involved in the regulation of β3-AR gene expression was verified taking advantage of the mouse model of oxygen-induced retinopathy (OIR), a model of retinal angiogenesis that is widely used as a paradigm to study angiogenesis in general [16]. This model is characterized by two phases: phase I or a “vaso-obliterative” phase coinciding with hyperoxia and phase II or a “vaso-proliferative” phase coinciding with hypoxia [17]. Therefore, thanks to the alternation of a period of hyperoxia and a period of hypoxia, this model is paramount to study the dependence of gene expression from oxygen levels and HIF-1 activity. Here, we first investigated whether β3-AR expression varies in concomitance with changing oxygen levels at different stages of OIR. Whether preventing hyperoxia-induced degradation of HIF-1α may influence β3-AR expression was also investigated. In order to verify the possibility of HIF-1 interaction with the β3-AR gene, a theoretical approach based on in silico predictive analysis was then performed to search for putative HBSs. As they may be located in genomic regions outside the promoter, as part of transcriptional enhancers [18,19,20], the DNA sequence upstream and inside the β3-AR gene was analyzed. In addition, docking analysis and molecular dynamics (MD) simulation were consequently used for reconstructing the complete structure of mouse HIF-1 and to evaluate its feasibility to tightly bind to specific nucleotide sequences as suggested by the prediction analysis. To demonstrate the direct physical interaction between HIF-1 and the potential HBSs, chromatin immunoprecipitation (ChIP)-qPCR experiments were then performed under hypoxic conditions and timely-dependent β3-AR mRNA levels were evaluated to check the possibility that β3-AR expression may be triggered by HIF-1 interaction with HBS.

## 2. Materials and Methods

### 2.1. Animals

C57BL/6J mice were purchased from Charles River Laboratories, Italia (Calco, Italy) and mated in our breeding colonies. Experimental animals were housed in a regulated environment (23 ± 1 °C, 50 ± 5% humidity) with a 12 h light/dark schedule. Overall, 162 mice (6 for each experimental group), either male or female, were used. Of them, 120 mice were used to compare β3-AR mRNA or protein levels between control (48) and OIR mice (72), without (36 mice) or with (36 mice) dimethyloxalylglycine (DMOG) administration; 42 mice, including 12 controls and 30 OIR, were used in ChIP-qPCR experiments and β3-AR mRNA determination. Animal studies were carried out in compliance with the recommendations in the Guide for the Care and Use of Laboratory Animals of the National Institutes of Health, the ARVO Statement for the Use of Animals in Ophthalmic and Vision Research, the Italian guidelines for animal care (DL 6/14) and the European Communities Council Directive (2010/63/UE). The experimental procedures were approved by the Ethical Committee in Animal Experiments of the University of Pisa (permit number 656/2018-PR, 3 September 2018). All efforts were made to reduce the animal suffering and the animal number based on the rules of replacement, refinement and reduction (the 3Rs).

### 2.2. OIR Model and DMOG Treatment

In the mouse model of OIR [17], litters of mouse pups with their nursing mothers were exposed to high oxygen concentration (75 ± 2%; hyperoxia) from postnatal day (PD) 7 to PD12 before returning to room air from PD12 to PD17 (normoxia sensed as relative hypoxia). During the exposure to hyperoxia, 36 mice were treated with DMOG, an inhibitor of prolyl hydroxylases that stabilizes HIF-1α also in the presence of oxygen by preventing its hydroxylation and the consequent degradation [21]. DMOG (Cayman Chemical, East Ellsworth, MI, USA) was dissolved in dimethyl sulfoxide, diluted in phosphate-buffered saline (PBS) at 1:100 and daily intraperitoneally injected between PD7 and PD12 at 200 mg/kg in line with previous studies of the mouse retina [22,23].

### 2.3. Western Blotting

After mice euthanasia, eyes were enucleated, and retinas were removed from the eyecups, snap frozen in liquid nitrogen, and stored at −80 °C. Six independent samples, each containing 2 retinas from 2 different mice, were used for each experimental condition. Samples were lysed with RIPA lysis buffer (50 mmol/L Tris, pH 7.4 containing 150 mmol/L NaCl, 1% Triton X-100, 1% sodium deoxycholate, 0.1% SDS, 5 mmol/L EDTA) containing proteinase and phosphatase inhibitor cocktails (Roche Applied Science, Indianapolis, IN, USA). Protein concentration was measured using the Micro BCA Protein Assay (Thermo Fisher Scientific, Waltham, MA, USA). For each sample, 30 µg of proteins were subjected to SDS-PAGE (4–20%; Bio-Rad Laboratories, Hercules, CA, USA). Gels were transblotted onto a nitrocellulose membrane (Bio-Rad Laboratories). Blots were blocked in 3% skim milk for 1 h at room temperature and then incubated overnight at 4 °C with the following primary antibodies: rabbit polyclonal against HIF-1α (ab2185; Abcam, Cambridge, UK; 1:500 dilution), rabbit polyclonal against VEGF (ab9570; Abcam; 1:1000 dilution), mouse monoclonal against β3-AR (sc-515763; Santa Cruz Biotechnologies, Santa Cruz, CA, USA; 1:200 dilution), mouse monoclonal against β-actin (A2228; Sigma Aldrich, St. Louis, MO, USA; 1:2500 dilution). After washing, membranes were incubated for 2 h at room temperature with appropriate HRP-conjugated anti-mouse or anti-rabbit secondary antibodies (1:5000 dilution). Blots were developed using Clarity Western enhanced chemiluminescence substrate (Bio-Rad Laboratories) and images were acquired using ChemiDoc XRS+ (Bio-Rad Laboratories). The quantification of Western blot bands has been performed with the automated software Image Lab 6.0 (Bio-Rad Laboratories) by considering the integrated optical density (OD) that derives from each pixel with given xy coordinates within the band area. The OD for each target protein was normalized to that of β-actin used as loading control.

### 2.4. Quantitative Real-Time PCR

Six independent samples for each experimental condition, each containing 2 retinas from 2 different mice, were used. Total RNA was extracted (RNeasy Mini Kit; Qiagen, Valencia, CA, USA), purified, resuspended in RNase-free water and quantified. First-strand cDNA was produced from 1 µg of total RNA (QuantiTect Reverse Transcription Kit; Qiagen). The SsoAdvanced Universal SYBR Green Supermix (Bio-Rad Laboratories) was used to perform real-time PCR amplifications on a CFX Connect Real-Time PCR detection system equipped with the software CFX manager (Bio-Rad Laboratories). Forward and reverse primers were chosen to hybridize to unique regions of the appropriate gene sequence. Their sequences are as follows: β3-AR forward, 5′-TCTCTGGCTTTGTGGTCGGA-3′; β3-AR reverse, 5′-GTTGGTTATGGTCTGTAGTCTCG-3′; Rpl13a forward, 5′-CCAGGTATACAAGCAGGTGTGCTC-3′; Rpl13a reverse, 5′-CATCATTAGGGCCATCCTGGAC-3′. Primer amplification efficiency was close to 100%. The target gene was run concurrently with Rpl13a, a stable housekeeping gene in the OIR model [24]. Samples were compared using the relative threshold cycle (Ct method) and expressed as fold increase. All reactions were run in triplicate.

### 2.5. Prediction of HBSs

Mouse β3-AR gene and its mRNA sequences were obtained from the NCBI Reference Sequence database (gene ID: 11556). The transcription start site (TSS) was mapped by DNA/RNA alignment using Clustal Omega online tool (https://www.ebi.ac.uk/Tools/msa/clustalo/; accessed on 12 January 2020) between genomic DNA and the longest β3-AR mRNA (misc_RNA (XR_004934751.1)) at the position 27230845 of the chromosome 8 (strand-). The DNA sequence of mouse chromosome 8 (UCSC Genome Browser Mouse (GRCm38/mm10)) was analyzed from the absolute position 27237610 to 27226612 to search for potential HBSs using the PROMO software online tool (http://alggen.lsi.upc.es/cgibin/promo_v3/promo/promoinit.cgi?dirDB=TF_8.3; accessed on 12 January 2020). To search for the core sequence ACGTG, which is highly conserved inside HBSs [25,26,27], the Fuzzy Search DNA on-line tool (https://www.genscript.com/sms2/fuzzy_search_dna.html; accessed on 12 January 2020) was used. A comparable evaluation was also performed for the human β3-AR gene (gene ID: 155) by analyzing the human chromosome 8 sequence (strand-) from the absolute position 37971666 to 37960995 (UCSC Genome Browser on Human (GRCh38/hg38)).

### 2.6. Computational Modeling of HIF-1α

The complete structure of the mouse HIF-1α was reconstructed starting from crystallographic data of protein fragments stored in both Uniprot (code Q61221) and Protein Data Bank (codes 4h6j) using Iterative Threading ASSEmbly Refinement (I-TASSER) [28]. In addition, ModLoop [29] and GalaxyLoop [30] were used for loop modeling and refinement, respectively. DISULFIND online server [31] was used to predict the disulfide bonding state of cysteines and their disulfide connectivity’s. The completely rebuilt HIF-1α structure was opportunely stabilized creating a simulation box of 22.2 × 18.02 × 18.12 nm, and neutralizing the net charge of the protein with 205,981 TIP3P water molecules, 605 Na^+^ ions and 572 Cl^−^ ions to reach a salt concentration of 0.15 M. The protein was minimized with 10,000 cycles steepest descent followed by 5000 steps conjugate gradient thus obtaining a convergence of maximum force to energy threshold of 1000 kJ/mol nm^2^. Then, HIF-1α was gradually accommodated within the aqueous environment using 6 equilibration steps; the Verlet cutoff scheme was used for neighbor searching [32], combined with particle mesh Ewald for electrostatics [33]. The cutoff for the Van der Waals forces calculation was settled to 1.2 nm with force smoothly switched to 0 (between 1.0 and 1.2 nm) generating the velocities at 310 K in constant temperature, constant volume (NVT) ensemble using a Maxwell distribution function with random seed (Berendsen thermostat; 2 simulation runs, 25 ps). Then, we shifted to constant temperature, constant pressure (NPT) ensemble maintaining the weak coupling also for pressure control (Berendsen barostat, isotropic conditions, 1 bar, time coupling 5 ps), maintained for 4 equilibration runs (50 ps). In the 200 ns production phase, we shifted to Nosé–Hoover [34] and Parrinello–Rhaman [35] algorithms for temperature control and pressure coupling, respectively; leapfrog algorithm and a time step of 0.002 ps were used. AMBER99-SB-ILDN force field as implemented in GROMACS 5.0.4 software package [36] was also used.

### 2.7. HIF-1/DNA Docking

HDOCK server for Protein/DNA hybrid docking (http://hdock.phys.hust.edu.cn/; accessed on 13 March 2020) was used to assess the configuration and the stability of HIF-1 with specific DNA sequences that were selected on the basis of the known minimum consensus sequences [37,38,39,40,41]. The corresponding HIF-1/DNA 3D structures were built up and minimized using AMBER18 software and AMBER force field [42,43].

### 2.8. HIF-1/DNA MD Simulation

The HIF-1/DNA models obtained from the docking approach underwent to 100 ns of MD simulation following the MD protocol described above for the HIF-1α stabilization. Each HIF-1/DNA model included 2 monomers of the entire HIF-1α protein (monomer-A and monomer-B) together with each of the 6 DNA segments containing predicted HBSs. Since the information about the sequence and the domains of HIF-1β are scarce and uncertain to operate an efficient modeling of the full protein structure, the homodimeric association of HIF-1α was considered instead of its heterodimeric form with HIF-1β. The accuracy of this approach was confirmed by the observation that the coordinates of the heterodimeric HIF-1α:HIF-1β DNA binding domain, as provided by 4zpr pdb structure [44], are maintained with maximum accuracy in the homodimeric form. These coordinates were therefore used to create the DNA-binding site and the overall configuration of the dimeric interface. The simulation box for each system was 20.8 × 20.8 × 20.8 nm to contain the whole HIF-1 omodimer. Water molecules and ions were opportunely added to solvate the systems and to reach the physiological salt condition. Appropriate optimal salt concentration (267,174 TIP3P water molecules followed by 843 Na^+^ ions and 755 Cl^−^ ions, on average) was then added to each model to neutralize the net charges of proteins and DNA.

With the aim to identify specific bonds formed with the nucleotide sequences, the contacts between HIF-1 and DNA structures were investigated along 200 ns MD simulation using *gmx mindist* gromacs tool, while the analysis of the simulation trajectories was performed by means of the VMD [45] and CHIMERA software [46].

### 2.9. Chromatin Immunoprecipitation-Quantitative Real-Time PCR (ChIP-qPCR)

ChIP-qPCR was performed in 6 independent samples using the high-sensitivity ChIP kit (ab185913; Abcam) according to the instructions provided by the manufacturer. Six independent samples for each experimental condition, each containing 2 retinas from two different mice, were used. Samples were homogenized and crosslinked using 1% formaldehyde, quenching the reaction with 1.25 M glycine. After centrifugation, the pellet was homogenized with the Working Lysis Buffer and added with the ChIP buffer provided in the kit. Chromatin was then sheared into fragments of about 300 bp by sonication, and fragmentation was verified by agarose gel electrophoresis. Immunoprecipitation was performed using a ChIP-grade antibody against HIF-1α (ab2185; Abcam; 1:500 dilution) or non-immune IgG as negative control. Immuno-complexes and small aliquots of lysate (for input controls) were treated with DNA Release Buffer and Proteinase K provided in the kit to reverse crosslinking and to purify DNA. The purified DNA was used as input sample in qPCR using primers (forward 5′-ATGCCTCCTCTGTCTGTGTG-3′; reverse 5′-ACTCGCCTCTCAAACAGTCA-3′) mapping the most probable HBS among the different HBSs as detected by PROMO software online tool (see above). The SsoAdvanced Universal SYBR Green Supermix (Bio-Rad Laboratories) and a CFX Connect Real-Time PCR detection system equipped with the software CFX manager (Bio-Rad Laboratories) were used. Fold enrichment was calculated through the Ct method by using a ratio of amplification efficiency of the ChIP sample over that of non-immune IgG (fold enrichment = 2^(IgG Ct – sample Ct)^).

### 2.10. Statistics

The Shapiro-Wilk test was used to verify the normal distribution of the data. One-way analysis of variance (ANOVA) followed by Tukey’s multiple comparison post-hoc test was used to evaluate statistical significance (Prism 8.0.2, GraphPad Software, Inc., San Diego, CA, USA). Differences with *p* < 0.05 were considered statistically significant. Data are plotted as means ± SEM of the reported *n* values. A priori power analysis using the software G*Power 3.0.10 (www.gpower.hhu.de; accessed on 4 December 2019) was carried out to determine the minimum number of animals necessary to obtain a statistical power of at least 0.80, with α = 0.05, when considering an expected large effect size.

## 3. Results

### 3.1. HIF-1-Regulated β3-AR Expression

**Figure 1:**
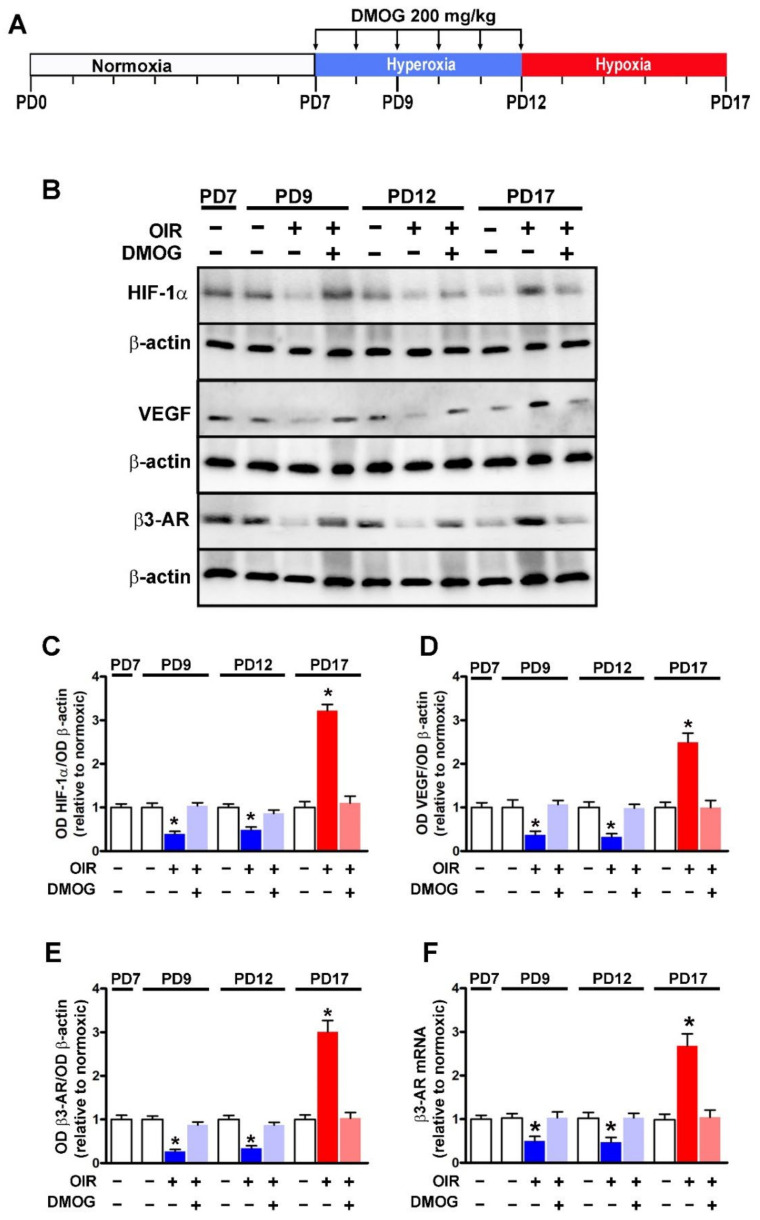
Effects of oxygen tension on retinal levels of HIF-1α, VEGF and β3-AR from PD7 to PD17. (**A**) Schematic diagram of the OIR model including DMOG administration daily from PD7 to PD12. (**B**) Representative blots showing protein levels of HIF-1α, VEGF and β3-AR as evaluated by Western blot in retinal extracts at different times from normoxic controls or OIR mice without or with DMOG administration. β-actin was used as the loading control. (**C**–**E**), Relative densitometric analyses of the protein levels of HIF-1α, VEGF and β3-AR. (**F**) Retinal mRNA levels of β3-AR at different times from controls or OIR mice untreated or treated with DMOG. * *p* < 0.05 vs. normoxic controls. One-way ANOVA followed by Tukey’s multiple comparison post-hoc test. Each histogram represents the mean ± SEM of data from 6 independent samples.

Retinal levels of HIF-1α, VEGF and β3-AR were determined at different time points of OIR (before the hyperoxic phase at PD7, during the hyperoxic phase at PD9, at the end of the hyperoxic phase at PD12 or at the end of the hypoxic phase at PD17). In order to assess whether oxygen may regulate β3-AR levels through HIF-1, protein levels of HIF-1α, VEGF and β3-AR were also determined in OIR mice that received DMOG, a chemical widely used as a HIF-1α stabilizer [21], administered daily during the hyperoxic phase (from PD7 to PD12; Figure 1A). Blots in Figure 1B are representative of the protein levels of HIF-1α, VEGF and β3-AR in the different experimental conditions. Densitometric analysis shown in Figure 1C-E demonstrates that proteins levels of HIF-1α, VEGF and β3-AR were downregulated during hyperoxia both at PD9 and PD12, while they became upregulated during hypoxia at PD17 compared with their relative levels as determined under normoxia. In respect to untreated mice, DMOG administration prevented downregulation of HIF-1α, VEGF and β3-AR in response to hyperoxia as well as their upregulation in response to hypoxia. The finding that the effects of hyperoxia or hypoxia on HIF-1α and VEGF were abnegated after DMOG administration is in line with previous results [22,47,48]. The additional finding that HIF-1α stabilization counteracts variations in protein levels of β3-AR in response to changing oxygen levels supports the hypothesis that oxygen regulates β3-AR expression through HIF-1. β3-AR dependence on HIF-1 was also supported by the fact that protein levels of β3-AR were found to mirror the levels of VEGF, a major oxygen-dependent HIF-1 transcript. Variations in β3-AR proteins in response to changing oxygen tension were found to correlate with the corresponding variations in β3-AR mRNA expression in OIR mice either untreated or treated with DMOG further supporting the possibility that β3-AR is a HIF-1 target gene (Figure 1F).

### 3.2. Potential HBSs as Revealed by DNA Prediction Analysis

**Figure 2:**
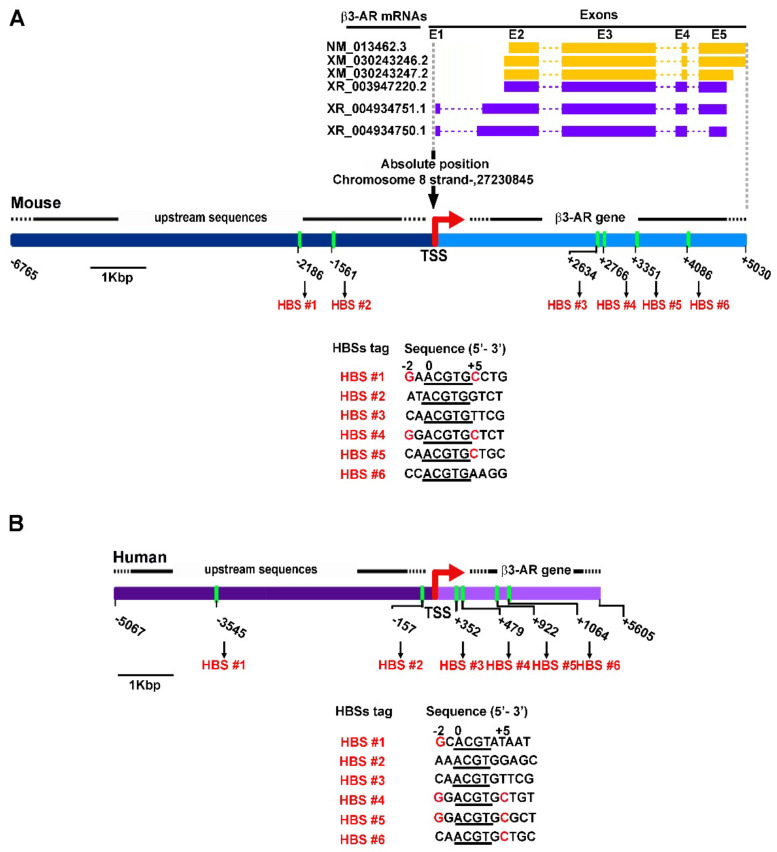
Schematic representation of mouse and human β3-AR genes including their upstream sequences. (**A**) In the mouse gene, 5 exons (E1–E5; solid boxes) and 4 introns (dashed lines) are depicted. They potentially express up to 6 different alternative mRNAs of which 3 codify for the canonical β3-AR protein (yellow mRNAs) while the other 3 for an alternative β3-AR protein with a different C-terminal sequence (purple mRNAs). The putative transcription-start site (TSS) is indicated by the red arrow. The positions of the 6 potential HBSs relative to the TSS are in green. All of them contain the minimal HBS consensus sequence (underlined sequence 5′-ACGTG-3′). (**B**) In the human gene, the positions of the 6 potential HBSs relative to the TSS are in green. All of them contain the minimal consensus sequence (underlined sequence 5′-ACGT-3′). The putative TSS is indicated by the red arrow. The highly conserved nucleotides G^−2^ and/or C^+5^ in the mouse and human HBSs sequence are highlighted in red.

The finding that β3-AR expression may depend on HIF-1 levels prompted us to verify whether potential HBSs can be present in the region upstream and inside the mouse and the human β3-AR gene (Figure 2). As reported by NCBI Reference Sequence Database and represented in Figure 2A, the mouse β3-AR gene contains 5 exons (E1-E5) that may potentially express up to 6 different alternative mRNAs. Of them, 3 codify for the canonical β3-AR protein (Figure 2A, green mRNAs) while the other 3 may be predicted to codify for an alternative β3-AR protein with a different C-terminal sequence (Figure 2A, purple mRNAs). Following DNA/RNA alignment between the genomic DNA and the longest β3-AR mRNA, the TSS was predicted to be localized at the absolute position 27230845 of the mouse chromosome 8. The TSS was taken as a reference for the localization of the potential HBBs. As HBSs can be localized at high distance with respect to the TSS [18] or even inside the transcriptional region [19,20], we searched for potential HBSs by analyzing the DNA sequence from the position −6765 upstream the TSS up to the position +4234 downstream the TSS using PROMO software [49,50] and searching for the minimal HBS consensus sequence ACGTG known to be strongly conserved at the HBS level [25,26,27] using the Fuzzy Search DNA tool. We found 6 different potential HBSs (Figure 2A, HBSs #1–6). HBSs #1, #4 and #5 contain the ACGTG core sequence and were directly predicted by PROMO, while HBSs #2, #3 and #6 contain ACGTG core sequence were not predicted by PROMO as potential HBSs but were identified by Fuzzy Search DNA tool. To complete this analysis, we evaluated the contribution of nucleotides additional to the ACGTG core sequence in all 6 putative HBSs by comparing their sequences with those previously characterized for a wide number of HIF-target genes [25,26,51]. We found that HBSs #1, #4 and #5 contain the G^−2^ and/or the C^+5^ nucleotides (red highlighted in Figure 2A) in respect to the A^0^CGTG core sequence, two nucleotides demonstrated an ability to increase the probability that HIF-1 binds to the HBS [25]. These nucleotides are absent at level of HBSs #2, #3 and #6, which contain only the core sequence. For these reasons HBSs #1, #4 and #5 were predicted as high-probability HBSs and, among them, the HBS #1 was the only one identified into the DNA region upstream the TSS at −2186, a position compatible with a classical transcriptional enhancer. As shown in Figure 2B, upstream and inside the human β3-AR gene, 6 HBSs were also predicted. Of these, HBSs #3–6 are localized inside the β3-AR gene, while HBSs #1 and #2 are localized upstream the putative TSS. In respect to the data reported for the mouse β3-AR gene, human HBSs #1, #4, #5 and #6 show the G^−2^ and/or the C^+5^ nucleotides (highlighted in red in Figure 2B) in addition to the conserved core sequence. Beside containing the G^−2^ nucleotide, the human HBS #1 was predicted at −3545, a position comparable to that of the mouse HBS #1.

### 3.3. Stability of the HIF-1α Protein Model

**Figure 3:**
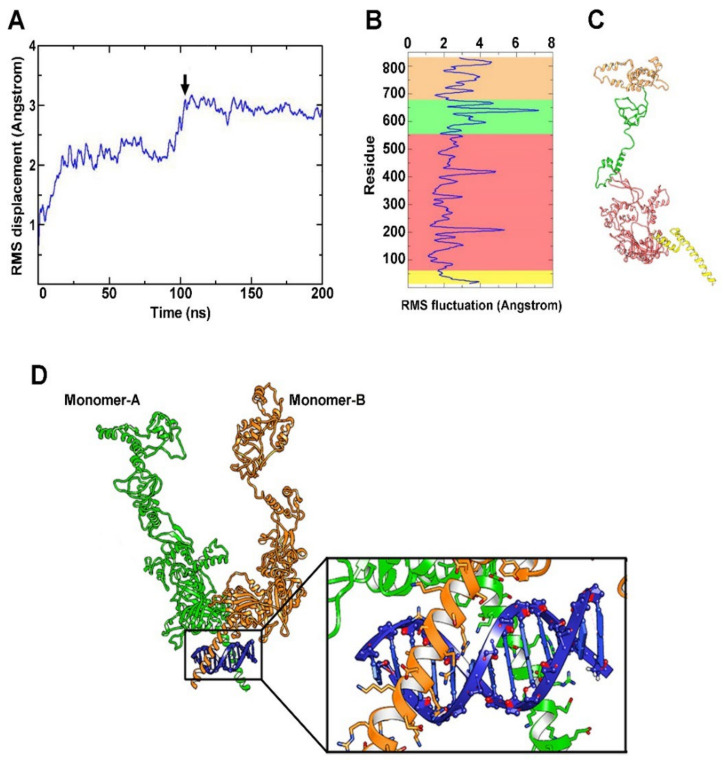
HIF-1α modeling and HIF-1/DNA docking. (**A**) Root-mean-square (RMS) displacement of protein backbone (black arrow indicates the time at which the stabilization of the protein structure occurs). (**B**) RMS fluctuation of aminoacid displacement relative to the starting structure and the principal domains of the HIF-1α protein, accordingly colored in (**C**). (**D**) HIF-1α protein modelized in its dimeric form showing the correct interaction with the DNA fragment. The two monomers are reported in green and orange respectively, while the DNA fragment is highlighted in blue. The binding site generated by dimerization is better shown in the focus section.

The stability of the rebuilt structure of HIF-1α has been monitored using the root-mean-square (RMS) deviation analysis, which represents the average displacement of the atoms over the simulation time relative to a reference structure. This type of analysis allows us to observe when the trajectories of displaced atoms plateau and converge towards an equilibrium state. As shown in Figure 3A, the HIF-1α protein model stabilizes after 100 ns and remains stable throughout 200 ns of MD simulation. To describe the structural mobility of HIF-1α protein, the RMS fluctuation analysis has been performed. It consists in a measurement of the displacement of aminoacids relative to the starting structure. The fluctuation degree of aminoacids plays a key role for specific biological function; for this reason the last 20 ns of MD simulation have been considered to define the RMS fluctuation profile. Figure 3B shows that different HIF-1α regions, as evidenced by the corresponding colors in Figure 3C, display a different fluctuation trend confirming the presence of flexible domains in the HIF-1α structure. Since the domains of HIF-1α dimerization and binding with DNA (Crystal Structure of the Heterodimeric HIF-1a:ARNT Complex with HRE DNA as determined by Protein Data Bank) are known to correspond to the first portion of the protein (i.e., the yellow domain in Figure 3C), HIF-1 in its dimeric form has been modeled using the coordinates of the heterodimeric HIF-1α:HIF-1β DNA binding domain as available from 4zpr pdb file [44]. Thereby, modeling of HIF-1β has been avoided because of the scarce information in terms of its aminoacid sequence and crystallographic structure. To confirm the reliability of the obtained dimeric interface, a comparison between the present model and the 4zpr interface has allowed us to demonstrate a close superimposition of the helices in the DNA binding domain (Figure S1).

After energy minimization, the final structure of HIF-1 in its dimeric form was recognized as the starting point for the subsequent investigation of affinity and specificity between HIF-1 and the different HBSs. In the representative model of Figure 3D, the correct interaction between the DNA binding portion of the HIF-1 dimer and the DNA fragment is shown.

### 3.4. Simulation of HIF-1/DNA Interaction and Binding Stability

**Figure 4:**
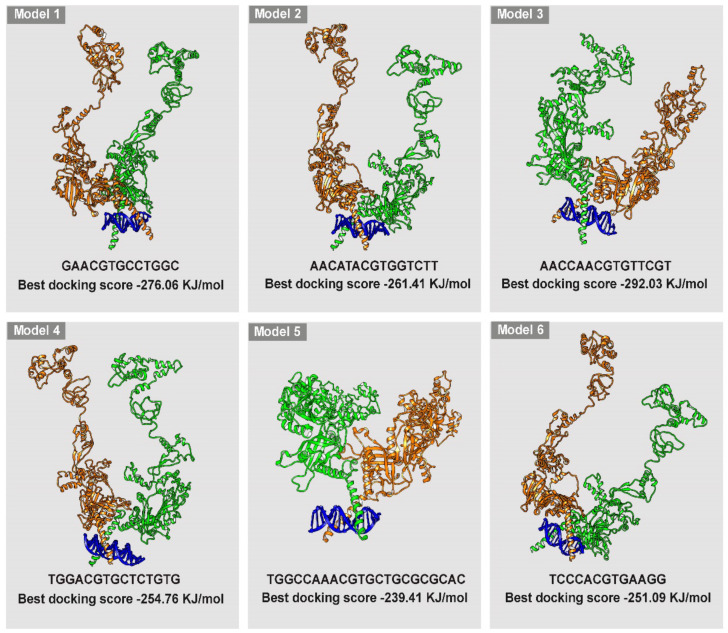
Graphical representation of the 6 HIF-1/DNA models displaying the 3D reconstruction of the HIF-1 docking to each of the nucleotide sequence, including HBS and flanking sequences, and their relative best docking score in kJ/mol.

The analysis of HIF-1/DNA docking identified 6 possible DNA segments (models 1–6) corresponding to the 6 HBSs found in the mouse β3-AR genes by computational analysis with additional flanking nucleotides upstream and downstream the HBS (model 1, GAACGTGCCTGGC, containing HBS #1; model 2, AACATACGTGGTCTT, containing HBS #2; model 3, AACCAACGTGTTCGT, containing HBS #3; model 4, TGGACGTGCTCTGTG, containing HBS #4; model 5, TGGCCAACGTGCTGCGCGCAC, containing HBS #5; model 6, TCCCACGTGAAGG, containing HBS #6). DNA sequences and reconstructions of the models 1–6 are represented in Figure 4. The results are reported according to the docking score for each simulated model. On the basis of the specificity of binding, which involves interactions with the nitrogen-containing bases and not merely with the phosphate skeleton, 2 models have been selected. In particular, among the 6 simulated models, model 1 and model 3 are those that show the highest affinity and specificity for HIF-1 (−276.06 and −292.03 kJ/mol, respectively). For both, the highest scored models bind to the DNA binding domain for HIF-1. The other models, both for their docking score (−261.41, −254.76, −239.97 and −251.09 kJ/mol for model 2, model 4, model 5 and model 6, respectively) and for the nature of their interactions do not appear as suitable candidates for HIF-1 binding. The characteristics of the docking models together with their 3-D structure including DNA binding are summarize in Figure 4.

**Figure 5:**
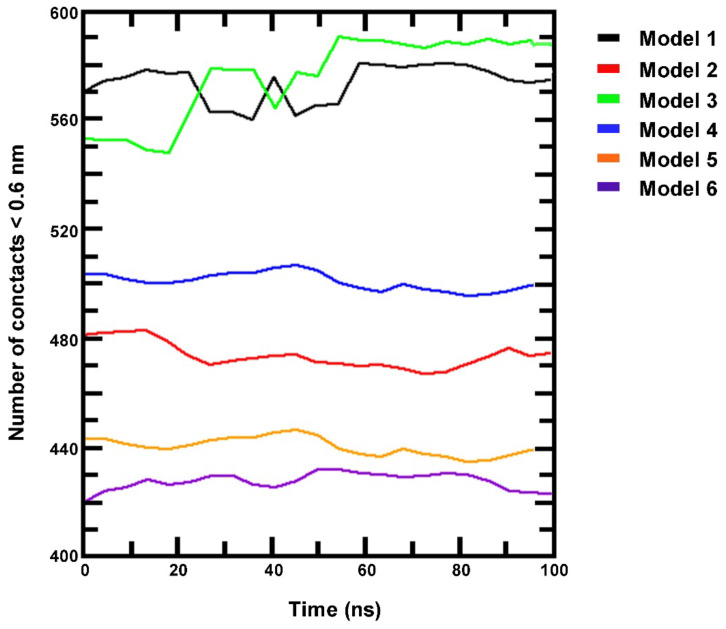
Direct interactions between HIF-1 and the 6 DNA fragments containing the HBSs as extrapolated from 100 ns molecular dynamics trajectory simulation, expressed as number of contacts within 0.6 nm distance between each other.

In order to make a definitive assessment of the binding stability, the selected models underwent 100 ns of MD simulation. Direct interactions between HIF-1 protein and DNA have been extrapolated from MD trajectories, and the results were plotted (Figure 5). The effective amount of the contacts was verified by considering the number of atom pairs that fall within a distance of 0.6 nm from each other. This is the maximum distance required for effective interactions between aminoacids and nucleotides. As shown in Figure 5, model 1 shows a high number of contacts, with an average value of 578 contacts in the last 10 ns. A similar trend was observed for model 3, with 584 contacts in the last 10 ns. In models 4 and 2, the average number of contacts in the last 10 ns are 504 and 476, respectively. Regarding models 5 and 6, the number of contacts in the last 10 ns is 436 and 428, respectively, indicating that their overall structures do not remain stable during MD simulation.

**Figure 6:**
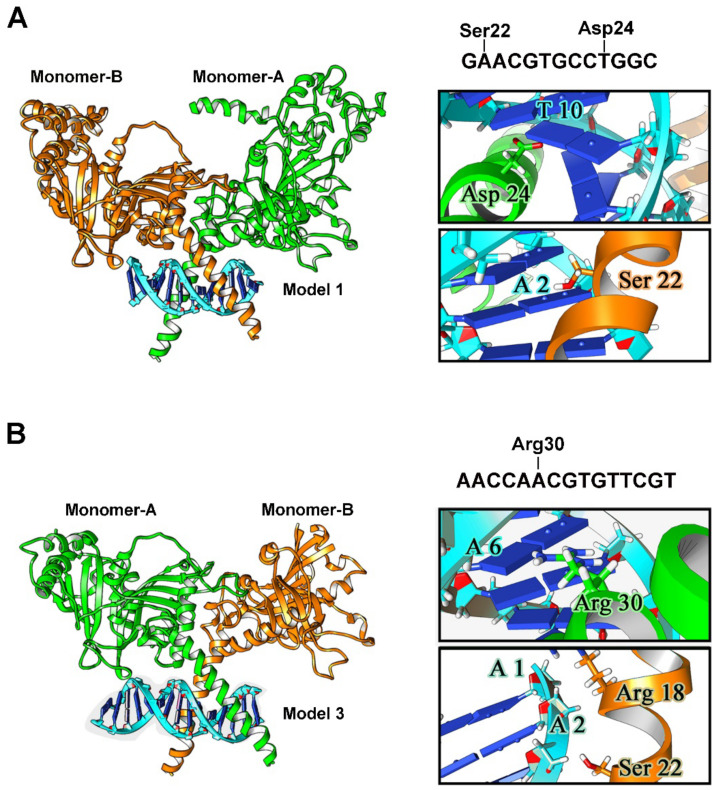
Docking analysis of model 1 and model 3. (**A**) HIF-1/HBS #1 best association complex: full structure and focus on HIF-1-DNA interactions (boxes). (**B**) HIF-1/HBS #3 best association complex: full structure and focus on HIF-1-DNA interactions (boxes).

Docking analysis shown in Figure 6A demonstrates that, in model 1, Asp24 of the monomer-A interacts with T in position 10 of the HBS #1while the hydroxyl group of Ser22 of the monomer-B interacts with A in position 2 of the flanking nucleotides upstream the HBS #1. On the contrary, in model 3 (Figure 6B), the observed interactions are less specific and only marginally involve the bases in the HBS #3. In particular, the 2 A in position 5 and 6, with A in position 6 being part of the HBS #3, interacts with Arg30 in monomer-A, whereas the other interactions (between A in position 1 and 2 with Arg18 and Ser22 in monomer-B, respectively) involve merely the phosphate skeleton and are electrostatic in nature. Therefore, model 3 although predicted with high affinity in its binding, displays Ser22 and Arg18 interactions mainly with the DNA phosphate skeleton thus resulting in low specificity and less stable binding over time. Model 1, instead, displays a wide number of interactions between aminoacidic residues of HIF-1 and the DNA bases contained in the HBS #1.

### 3.5. HIF-1/HBS #1 Interaction and β3-AR Gene Expression

**Figure 7:**
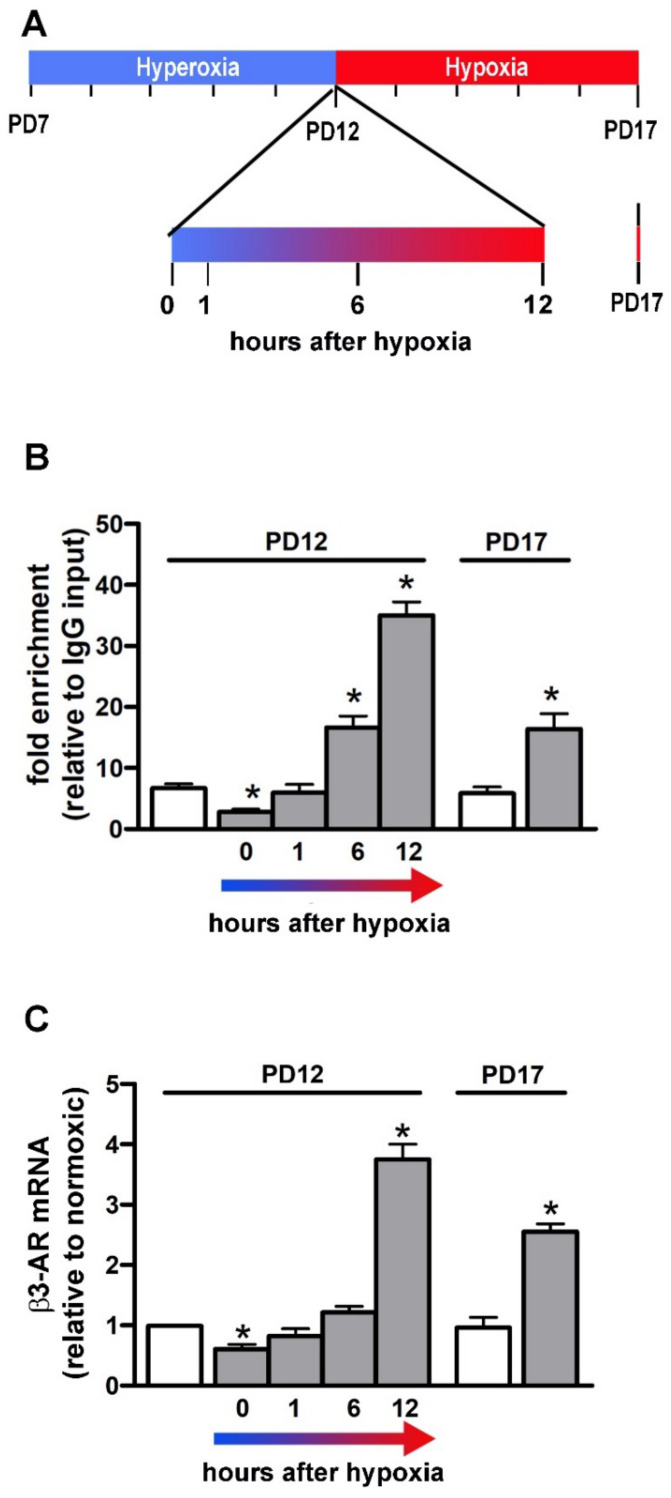
HIF-1α interaction with HBS #1 and corresponding β3-AR gene expression at PD12 (from 0 to 12 h of hypoxia) or at PD17. (**A**) Schematic diagram of the OIR model pointing to the specific times under analysis. (**B**) Data from HIF-1α chromatin immunoprecipitation and HBS #1-specific qPCR (ChIP-qPCR) represented as fold enrichment relative to IgG input. (**C**) Corresponding levels of β3-AR mRNA. White bars represent data from retinas of normoxic controls while grey bars represent data from hypoxic mice. One-way ANOVA followed by Tukey’s multiple comparison post-hoc test. Each histogram represents the mean ± SEM of data from 6 independent samples. * *p* < 0.05 vs. normoxic controls (*n* = 6 samples).

Overall, docking results and MD simulation point to HBS #1 as the HBS to which HIF-1 is most likely to bind. To explore the interaction between HIF-1α and HBS #1, HIF-1α-specific ChIP-qPCR analysis was performed in the mouse retina at PD12 (immediately after the end of hyperoxia as well as at 1, 6 and 12 h after exposure to relative hypoxia) and at PD17 (Figure 7A). These time points were identified as appropriate for the effective detection of HIF-1-dependent VEGF transcription in the OIR model [52]. As shown in Figure 7B, the interactions between HIF-1 and HBS #1, as determined by HBS #1 enrichment relative to the IgG input, are drastically lower at the end of hyperoxia (PD12, time 0) than in normoxic controls to then gradually increase from 1 to 12 h of hypoxia when about 35-fold enrichment is reached. At that time, the HBS #1 enrichment is about 6-fold higher than in normoxic controls. At 5 days of hypoxia (PD17), the interactions between HIF-1 and HBS #1 are lower than after 12 h of hypoxia, but similar to those measured after 6 h of hypoxia. At PD17, the interactions between HIF-1 and HBS #1 are about 3-fold higher in respect to normoxic controls. β3-AR gene expression was also assessed in order to evaluate whether HIF-1/HBS #1 interaction would be correlated with β3-AR gene expression. As shown in Figure 7C, β3-AR mRNA levels gradually increase from 0 to 12 h of hypoxia in close similarity with the HIF-1/HBS #1 interaction profile. In respect to normoxic controls, mRNA levels of β3-AR are downregulated at the end of hyperoxia to then time-dependently increase with a peak at 12 h of hypoxia. At PD 17, β3-AR mRNA levels are higher than in normoxic controls but lower than after 12 h of hypoxia.

## 4. Discussion

Maintaining oxygen homeostasis is crucial for oxygen-dependent metabolism, and exposure to low oxygen tension needs to be faced by adaptive responses, many of which are triggered by HIF-1. Here, we demonstrate that an active HIF-1 binding site, the HBS #1, is present upstream the mouse β3-AR TSS and that the physical interaction between HIF-1 and HBS #1 is correlated with β3-AR transcription. This suggests that HBS #1 might be a transcriptional regulator of β3-AR gene in the mouse retina thus playing a central role in determining β3-AR expression when retinal oxygen tension changes.

In a recent review, the possibility that β3-AR upregulation may be viewed as a general marker of hypoxic conditions has been discussed [13]. In solid tumors, in particular, β3-AR upregulation occurs in response to tumor cell proliferation leading to oxygen and nutrients deprivation that drives the activation of key transcription factors regulating a large panel of genes that allows the tumor cells to escape from an oxygen-deprived environment. Infantile hemangioma (a benign tumor of childhood), for instance, and a wide range of malignant tumors, which share dedifferentiation and hypoxia as main pathological features are characterized by drastic β3-AR upregulation. Upregulation of β3-AR mRNA and protein also characterizes additional ischemic conditions as for instance the failed heart in which β3-AR ligands have been demonstrated to ameliorate the pathological signs of cardiac injury [12,53,54]. The present findings that β3-ARs are upregulated in the retina in response to hypoxia in concomitance with HIF-1α is in line with previous findings indicating the possibility that increased levels of β3-AR would be coupled to the retinal angiogenic response [15,55]. Upregulated levels of HIF-1α and β3-AR would trigger a compensatory response by promoting VEGF accumulation causing the proliferation of new vessels and the vasodilation of existing vessels. The additional finding that retinal β3-AR is downregulated by hyperoxia in concomitance with lowered levels of HIF-1α and VEGF opens the question of whether β3-AR downregulation would be involved in retinal vessel regression in response to high oxygen tension.

β3-AR downregulation at birth has been recently demonstrated in the ductus arteriosus (DA), a fetal blood vessel connecting the aorta to the pulmonary artery. DA remains open during the hypoxic intrauterine environment, whereas it normally closes soon after birth in concomitance with a drastic decrease of β3-AR levels suggesting the possibility that β3-AR participates in DA patency [56]. Additional evidence from peripheral blood mononuclear cells is indicative of β3-AR downregulation during the switch from hypoxia to normoxia sensed as hyperoxia [57]. The present finding that the expression profile of retinal β3-ARs depends on changing oxygen tension similarly to HIF-1α and VEGF is intriguing if one considers that the panel of genes transactivated by HIF-1 plays a central role in cellular adaptation to oxygen deprivation [58].

Although receptor levels may depend on transcriptional and/or post-transcriptional mechanisms, the present finding that β3-AR mRNA is accordingly regulated by oxygen levels further supports the possibility that changing protein levels might be due to variations in β3-AR gene expression. The additional finding that abolishing hyperoxia-induced HIF-1α degradation prevents oxygen-dependent modulation of both β3-AR and VEGF reinforces the possibility that β3-AR, similar to VEGF, behaves as a HIF-1 target gene. However, the definitive establishment of HIF-1 role in the transcriptional regulation of β3-AR would imply the identification of a functional HRE with which HIF-1 could physically interact. In this respect, a previous study failed to demonstrate the presence of HRE consensus sites in the promoter/enhancer region of the mouse β3-AR gene suggesting that hypoxia-induced β3-AR upregulation derives exclusively from the post-transcriptional regulation of β3-AR mRNA and/or from the stabilization of β3-AR protein [59]. On the other hand, the high variability of the flanking sequences of the HRE might limit its determination which, in contrast, might be facilitated by the identification of the highly conserved cores i.e., the HBSs. Therefore, we investigated the presence of HBSs by analyzing the chromosome 8 sequences spanning upstream and inside the mouse β3-AR gene since potential HBSs can be distant from the TSS, thus increasing the probability to find them. The present results reveal 6 different potential HBSs suggesting the presence of multiple sites of interaction through which HIF-1 could actually bind to mouse β3-AR gene to modulate its expression. In particular, HBS #1 and HBS #2 are localized upstream the TSS in a region containing other established β3-AR gene enhancers [60] thus increasing the probability for these sites to represent functional HBSs. HIF-1 binding to HBS#1 is further supported by the fact that the nucleotides which are present upstream and downstream the minimal consensus core have been demonstrated to play an important role in the interaction between HIF-1 and HBSs [25]. The additional finding that human and mouse HBS #1 are located in corresponding regions further reinforces the possibility that HBS #1 is the best candidate for potential binding to HIF-1.

Still, the HBS consensus sequence is highly abundant across the genome, but less than 1% of potential sites are actually bound by HIF-1 as demonstrated by genome-wide analyses of HIF chromatin occupancy [25,26,61,62]. Therefore, a computational analysis allowing the simulation of the HIF-1 binding affinity and specificity has been used to assess the actual possibility of HIF-1 physical interaction with each of the identified HBSs. To this aim, the 3D structure of HIF-1α has been reconstructed using the full HIF-1α aminoacidic sequence and crystallographic data relative to HIF-1α fragments. As shown by the present results, the build structure reaches a stable conformation at 100 ns after an initial rise that is indicative of the equilibration of the system. The additional presence of flexible domains confirms that the HIF-1α structure is stable, but with the flexibility needed to allow for DNA binding. HIF-1 in its dimeric form has been approximated to HIF-1α homodimer instead of a HIF-1α/HIF-1β heterodimer since HIF-1α and HIF-1β show similar flexibility patterns in regions close to the dimerization portion [44]. The superimposition between the homodimer and the dimer interface in the 4zpr structure shows that the present model preserves the exact coordinates to generate the DNA binding domain. The analysis of HIF-1/DNA docking has allowed the identification of 6 possible models, each of them including the DNA sequence corresponding to each HBS sequence plus upstream and downstream additional nucleotides. A similar analysis has been previously performed for HIF-1/HBSs docking in the VEGF gene promoter [63]. Among the 6 models identified here, model 1, including HBS #1, and model 3, including HBS #3, have been predicted as the best models based on both docking score and number of contacts. The fact that model 3 displays HIF-1/DNA interactions mainly based on contacts of aminoacidic residues with the phosphate skeleton suggests that, despite the high docking score and the high number of contacts, this model would be devoid of the specificity required for an effective HIF-1 binding. Conversely, together with a high docking score and a high number of contacts, in model 1, HIF-1 interacts exclusively with nucleotides within the consensus sequence suggesting both affinity and specificity compatible with an actual HIF-1 binding.

Overall, the integration of data deriving from the bioinformatic analysis about the localization of HBS #1 and the computational analysis regarding its compatibility for HIF-1 indicate HBS #1 as the most suitable site for HIF-1 binding to the β3-AR gene.

The validation of the actual HIF-1 physical interaction with the HBS #1 derives from the ChIP-qPCR analysis, which definitively allows us to demonstrate HBS #1 as an effective HIF-1 binding site. In particular, the present findings show a time-dependent increment in HIF-1/HBS #1 physical interaction during the first hours of hypoxia, similar to that demonstrated for the VEGF gene in the OIR model [52]. Although the presence of a specific site for the HIF-1 binding confers hypoxic inducibility to the β3-AR gene, additional conditions such as the recruitment of coactivators or epigenetic modifications may be required to assemble a fully functional transcription complex [64,65]. Therefore, the physical interaction of HIF-1 with the HBS #1 does not necessarily imply an actual transcriptional regulation of the β3-AR gene by HIF-1. However, the close correlation between the time-dependent profile of the HIF-1/HBS #1 interaction and the corresponding β3-AR mRNA expression indicates that, under hypoxia, the HIF-1 binding to the HBS #1 may effectively induce the β3-AR gene expression and the consequent increase of β3-AR protein levels.

HIF-1-mediated β3-AR expression opens the question of whether this event constitutes only a marker of oxygen levels or is functionally related to hypoxia-associated events such as angiogenesis. In this respect, the possibility exists that increased β3-AR expression may play a functional role during the early phase of oxygen deprivation by promoting the angiogenic drive in support of suffering retinal cells. Although β3-AR’s role in retinal angiogenesis remains so far to be clarified, preliminary findings are indicative of β3-AR’s contribution to massive vessel proliferation in response to hypoxia. In particular, results obtained from retinal explants demonstrate that a β3-AR blockade or silencing prevents hypoxia-induced VEGF upregulation [66]. In addition, in β1/β2-AR knock out mice undergoing the OIR protocol, β3-AR agonism results in a dramatic increase in VEGF accumulation in concomitance with retinal vessel proliferation in response to hypoxia [67]. However, the finding that β3-AR antagonism does not influence hypoxia-associated increase in VEGF levels and retinal vascularization [24], although in contrast with a possible role of β3-AR in angiogenesis, may be due to the fact that many of β3-AR antagonists bind β3-AR with a low affinity and specificity [68,69]. In contrast to β3-AR antagonism, selective agonists are efficiently used for the treatment of overactive bladder in which β3-AR activation leads to myorelaxant effects and increases bladder capacity [70]. In addition, the fact that β3-AR agonism causes angiogenesis and vasodilation in the ischemic heart [14,71] led to new pilot clinical trials assessing β3-AR agonists as promising therapeutic targets for the treatment of heart failure [12]. In the retina, β3-AR overstimulation during hyperoxia might serve to prevent vessel regression thus limiting vessel proliferation in response to hypoxia similarly to that which has already been observed through preventing HIF-1α degradation [47,48], but without the complication derived from the activation of a vast array of genes.

Major constraints to the identifications of β3-AR’s role in ophthalmic diseases originate from the scarce data on β3-AR expression and function in the posterior segment of the human eye. Although the role of β3-ARs as a therapeutic target in ophthalmological diseases has been questioned [72], β3-AR involvement in the control of cell proliferation, migration, invasion and elongation has been demonstrated in human retinal and choroidal endothelial cells. In particular, β3-AR are expressed by human retinal endothelial cells where their activation promotes cell migration and proliferation both of which characterize vascular responses to altered sympathetic nerve activity [73]. In human choroidal endothelial cells, β3-AR activation contributes to cell invasion, proliferation and elongation thus suggesting β3-AR involvement in ocular diseases characterized by choroidal neovascularization [74]. These findings have been confirmed by recent data of Topcuoglu and Aslan [75] who have demonstrated that in patients with an overactive bladder, routinary treatment with mirabegron significantly increases choroidal vascular parameters suggesting that β3-ARs may have a role in eye diseases associated with choroidal vascularization.

## 5. Conclusions

Reduced oxygen availability to retinal cells may lead to the progression of hypoxia-induced proliferative diseases in which HIF-1 upregulation plays a major role. Although inhibiting HIF-1 can be considered as an effective treatment of oxygen-dependent ocular diseases, the use of most HIF-1 inhibitors is still debated because of their side effects and toxicities. In contrast, therapies related to HIF-1 target genes may have remarkable impact on counteracting hypoxia-related pathologies as it occurs for anti-VEGF therapies. Here, we suggest the possibility that β3-AR transcription may depend on HIF-1 thus supporting β3-AR involvement in HIF-1-mediated responses to hypoxia. In this respect, HIF-1 would transactivate both VEGF and β3-AR, which has been shown to regulate VEGF levels in response to low oxygen tension. This supports the possibility that β3-AR participates in the angiogenic response to hypoxia.

In summary, the present study shows an increased β3-AR expression in the hypoxic retina in which HIF-1 has been demonstrated to directly bind to the β3-AR enhancer region thus up-regulating β3-AR at the transcriptional level. Future studies will be required to clarify β3-AR’s role in hypoxia-driven angiogenesis to lay the ground for novel treatments of proliferative retinal diseases.

## Data Availability

The data presented in this study are available on request from the corresponding author.

## Supplementary Materials

The following supporting information can be downloaded at: https://www.mdpi.com/article/10.3390/cells11081271/s1, Figure S1: Close superimposition of the heterodimeric interface from 4zpr (orange) with the HIF1α homodimer model (green): full structure and focus on DNA binding domain (boxes).

## References

[1] Semenza G.L., Wang G.L. (1992). A nuclear factor induced by hypoxia via de novo protein synthesis binds to the human erythropoietin gene enhancer at a site required for transcriptional activation. Mol. Cell. Biol..

[2] Kaluz S., Kaluzová M., Stanbridge E.J. (2008). Regulation of gene expression by hypoxia: Integration of the HIF-transduced hypoxic signal at the hypoxia-responsive element. Clin. Chim. Acta.

[3] Hammarlund E.U., Flashman E., Mohlin S., Licausi F. (2020). Oxygen-sensing mechanisms across eukaryotic kingdoms and their roles in complex multicellularity. Science.

[4] Eelen G., Treps L., Li X., Carmeliet P. (2020). Basic and Therapeutic Aspects of Angiogenesis Updated. Circ. Res..

[5] Usui Y., Westenskow P.D., Murinello S., Dorrell M.I., Scheppke L., Bucher F., Sakimoto S., Paris L.P., Aguilar E., Friedlander M. (2015). Angiogenesis and Eye Disease. Annu. Rev. Vis. Sci..

[6] Casini G., Monte M.D., Fornaciari I., Filippi L., Bagnoli P. (2014). The β-adrenergic system as a possible new target for pharmacologic treatment of neovascular retinal diseases. Prog. Retin. Eye Res..

[7] Monte M.D., Martini D., Latina V., Pavan B., Filippi L., Bagnoli P. (2012). Beta-Adrenoreceptor Agonism Influences Retinal Responses to Hypoxia in a Model of Retinopathy of Prematurity. Investig. Opthalmol. Vis. Sci..

[8] Emorine L.J., Marullo S., Briend-Sutren M.-M., Patey G., Tate K., Delavier-Klutchko C., Strosberg A.D. (1989). Molecular Characterization of the Human beta 3-Adrenergic Receptor. Science.

[9] Calvani M., Cavallini L., Tondo A., Spinelli V., Ricci L., Pasha A., Bruno G., Buonvicino D., Bigagli E., Vignoli M. (2018). β3-Adrenoreceptors Control Mitochondrial Dormancy in Melanoma and Embryonic Stem Cells. Oxidative Med. Cell. Longev..

[10] Uhlén M., Fagerberg L., Hallström B.M., Lindskog C., Oksvold P., Mardinoglu A., Sivertsson Å., Kampf C., Sjöstedt E., Asplund A. (2015). Tissue-Based Map of the Human Proteome. Science.

[11] Dal Monte M., Calvani M., Cammalleri M., Favre C., Filippi L., Bagnoli P. (2019). β-Adrenoceptors as drug targets in melanoma: Novel preclinical evidence for a role of β3-adrenoceptors. Br. J. Pharmacol..

[12] Michel L.Y.M., Farah C., Balligand J.-L. (2020). The Beta3 Adrenergic Receptor in Healthy and Pathological Cardiovascular Tissues. Cells.

[13] Filippi L., Pini A., Cammalleri M., Bagnoli P., Dal Monte M. (2022). β3-Adrenoceptor, a novel player in the round-trip from neonatal diseases to cancer: Suggestive clues from embryo. Med. Res. Rev..

[14] Dessy C., Moniotte S., Ghisdal P., Havaux X., Noirhomme P., Balligand J. (2004). Endothelial Beta3-Adrenoceptors Mediate Vasorelaxation of Human Coronary Microarteries Through Nitric Oxide and Endothelium-Dependent Hyperpolarization. Circulation.

[15] Ristori C., Filippi L., Dal Monte M., Martini D., Cammalleri M., Fortunato P., la Marca G., Fiorini P., Bagnoli P. (2011). Role of the Adrenergic System in a Mouse Model of Oxygen-Induced Retinopathy: Antiangiogenic Effects of beta-Adrenoreceptor Blockade. Investig. Opthalmol. Vis. Sci..

[16] Selvam S., Kumar T., Fruttiger M. (2018). Retinal vasculature development in health and disease. Prog. Retin. Eye Res..

[17] Smith L.E.H., Wesolowski E., McLellan A., Kostyk S.K., D’Amato R.J., Sullivan R., D’Amore P.A. (1994). Oxygen-induced retinopathy in the mouse. Investig. Ophthalmol. Vis. Sci..

[18] Orlando I.M., Lafleur V.N., Storti F., Spielmann P., Crowther L., Santambrogio S., Schödel J., Hoogewijs D., Mole D.R., Wenger R.H. (2019). Distal and proximal hypoxia response elements cooperate to regulate organ-specific erythropoietin gene expression. Haematologica.

[19] Krueger K., Catanese L., Sciesielski L., Kirschner K., Scholz H. (2019). Deletion of an intronic HIF-2α binding site suppresses hypoxia-induced WT1 expression. Biochim. Biophys. Acta Gene Regul. Mech..

[20] Binó L., Procházková J., Radaszkiewicz K.A., Kučera J., Kudová J., Pacherník J., Kubala L. (2017). Hypoxia favors myosin heavy chain beta gene expression in an Hif-1alpha-dependent manner. Oncotarget.

[21] Strowitzki M.J., Cummins E.P., Taylor C.T. (2019). Protein Hydroxylation by Hypoxia-Inducible Factor (HIF) Hydroxylases: Unique or Ubiquitous?. Cells.

[22] Cammalleri M., Monte M.D., Locri F., Pecci V., De Rosa M., Pavone V., Bagnoli P. (2019). The urokinase-type plasminogen activator system as drug target in retinitis pigmentosa: New pre-clinical evidence in the rd10 mouse model. J. Cell. Mol. Med..

[23] Cammalleri M., Monte M.D., Amato R., Lapi D., Bagnoli P. (2020). Novel Insights into Beta 2 Adrenergic Receptor Function in the rd10 Model of Retinitis Pigmentosa. Cells.

[24] Martini D., Monte M.D., Ristori C., Cupisti E., Mei S., Fiorini P., Filippi L., Bagnoli P. (2011). Antiangiogenic effects of β2-adrenergic receptor blockade in a mouse model of oxygen-induced retinopathy. J. Neurochem..

[25] Schödel J., Oikonomopoulos S., Ragoussis J., Pugh C.W., Ratcliffe P.J., Mole D.R. (2011). High-resolution genome-wide mapping of HIF-binding sites by ChIP-seq. Blood.

[26] Mole D.R., Blancher C., Copley R.R., Pollard P.J., Gleadle J.M., Ragoussis J., Ratcliffe P.J. (2009). Genome-wide Association of Hypoxia-inducible Factor (HIF)-1α and HIF-2α DNA Binding with Expression Profiling of Hypoxia-inducible Transcripts. J. Biol. Chem..

[27] Kaluz S., Kaluzová M., Stanbridge E.J. (2008). Rational design of minimal hypoxia-inducible enhancers. Biochem. Biophys. Res. Commun..

[28] Yang J., Yan R., Roy A., Xu D., Poisson J., Zhang Y. (2015). The I-TASSER Suite: Protein structure and function prediction. Nat. Methods.

[29] Fiser A., Sali A. (2003). ModLoop: Automated modeling of loops in protein structures. Bioinformatics.

[30] Park H., Lee G.R., Heo L., Seok C. (2014). Protein Loop Modeling Using a New Hybrid Energy Function and Its Application to Modeling in Inaccurate Structural Environments. PLoS ONE.

[31] Ceroni A., Passerini A., Vullo A., Frasconi P. (2006). DISULFIND: A disulfide bonding state and cysteine connectivity prediction server. Nucleic Acids Res..

[32] Páll S., Hess B. (2013). A flexible algorithm for calculating pair interactions on SIMD architectures. Comput. Phys. Commun..

[33] Darden T., York D., Pedersen L. (1993). Particle mesh Ewald: An *N*⋅log(*N*) method for Ewald sums in large systems. J. Chem. Phys..

[34] Hoover W.G. (1985). Canonical dynamics: Equilibrium phase-space distributions. Phys. Rev. A.

[35] Parrinello M., Rahman A. (1981). Polymorphic transitions in single crystals: A new molecular dynamics method. J. Appl. Phys..

[36] Abraham M.J., Murtola T., Schulz R., Páll S., Smith J.C., Hess B., Lindahl E. (2015). GROMACS: High performance molecular simulations through multi-level parallelism from laptops to supercomputers. SoftwareX.

[37] Yan Y., Tao H., He J., Huang S.-Y. (2020). The HDOCK server for integrated protein-protein docking. Nat. Protoc..

[38] Yan Y., Zhang D., Zhou P., Li B., Huang S.-Y. (2017). HDOCK: A web server for protein-protein and protein-DNA/RNA docking based on a hybrid strategy. Nucleic Acids Res..

[39] Yan Y., Wen Z., Wang X., Huang S.-Y. (2017). Addressing recent docking challenges: A hybrid strategy to integrate template-based and free protein-protein docking. Proteins Struct. Funct. Bioinform..

[40] Huang S.-Y., Zou X. (2014). A knowledge-based scoring function for protein-RNA interactions derived from a statistical mechanics-based iterative method. Nucleic Acids Res..

[41] Huang S.-Y., Zou X. (2008). An iterative knowledge-based scoring function for protein-protein recognition. Proteins Struct. Funct. Bioinform..

[42] Case D.A., Ben-Shalom I.Y., Brozell S.R., Cerutti D.S. (2018). AMBER 2018.

[43] Wang J., Wolf R.M., Caldwell J.W., Kollman P.A., Case D.A. (2004). Development and testing of a general amber force field. J. Comput. Chem..

[44] Wu D., Potluri N., Lu J., Kim Y., Rastinejad F. (2015). Structural integration in hypoxia-inducible factors. Nature.

[45] Humphrey W., Dalke A., Schulten K. (1996). VMD: Visual molecular dynamics. J. Mol. Graph..

[46] Pettersen E.F., Goddard T.D., Huang C.C., Couch G.S., Greenblatt D.M., Meng E.C., Ferrin T.E. (2004). UCSF Chimera-a visualization system for exploratory research and analysis. J. Comput. Chem..

[47] Hoppe G., Yoon S., Gopalan B., Savage A.R., Brown R., Case K., Vasanji A., Chan E.R., Silver R.B., Sears J.E. (2016). Comparative systems pharmacology of HIF stabilization in the prevention of retinopathy of prematurity. Proc. Natl. Acad. Sci. USA.

[48] Sears J.E., Hoppe G., Ebrahem Q., Anand-Apte B. (2008). Prolyl hydroxylase inhibition during hyperoxia prevents oxygen-induced retinopathy. Proc. Natl. Acad. Sci. USA.

[49] Messeguer X., Escudero R., Farre D., Núñez O., Martınez J., Albà M. (2002). PROMO: Detection of known transcription regulatory elements using species-tailored searches. Bioinformatics.

[50] Farré D., Roset R., Huerta M., Adsuara J.E., Roselló L., Albà M.M., Messeguer X. (2003). Identification of patterns in biological sequences at the ALGGEN server: PROMO and MALGEN. Nucleic Acids Res..

[51] Ortiz-Barahona A., Villar D., Pescador N., Amigo J., Del Peso L. (2010). Genome-wide identification of hypoxia-inducible factor binding sites and target genes by a probabilistic model integrating transcription-profiling data and in silico binding site prediction. Nucleic Acids Res..

[52] Pisani F., Cammalleri M., Monte M.D., Locri F., Mola M.G., Nicchia G.P., Frigeri A., Bagnoli P., Svelto M. (2017). Potential role of the methylation of VEGF gene promoter in response to hypoxia in oxygen-induced retinopathy: Beneficial effect of the absence of AQP. J. Cell. Mol. Med..

[53] Balligand J.-L. (2016). Cardiac salvage by tweaking with beta-3-adrenergic receptors. Cardiovasc. Res..

[54] Michel L.Y.M., Balligand J.-L. (2016). New and Emerging Therapies and Targets: Beta-3 Agonists. Heart Failure.

[55] Chen J., Joyal J.-S., Hatton C.J., Juan A., Pei D.T., Hurst C.G., Xu D., Stahl A., Hellström A., Smith L.E.H. (2012). Propranolol Inhibition of β-Adrenergic Receptor Does Not Suppress Pathologic Neovascularization in Oxygen-Induced Retinopathy. Investig. Opthalmol. Vis. Sci..

[56] Pini A., Fazi C., Nardini P., Calvani M., Fabbri S., Guerrini A., Forni G., La Marca G., Rosa A.C., Filippi L. (2020). Effect of Beta 3 Adrenoreceptor Modulation on Patency of the Ductus Arteriosus. Cells.

[57] Calvani M., Bruno G., Dal Monte M., Nassini R., Fontani F., Casini A., Cavallini L., Becatti M., Bianchini F., De Logu F. (2019). β3-Adrenoceptor as a potential immuno-suppressor agent in melanoma. Br. J. Pharmacol..

[58] Dengler V.L., Galbraith M., Espinosa J.M. (2014). Transcriptional regulation by hypoxia inducible factors. Crit. Rev. Biochem. Mol. Biol..

[59] Monte M.D., Evans B.A., Arioglu-Inan E., Michel M.C. (2020). Upregulation of β3-adrenoceptors—a general marker of and protective mechanism against hypoxia?. Naunyn-Schmiedeberg’s Arch. Pharmacol..

[60] Dixon T.M., Daniel K.W., Farmer S., Collins S. (2001). CCAAT/Enhancer-binding Protein α Is Required for Transcription of the beta 3-Adrenergic Receptor Gene during Adipogenesis. J. Biol. Chem..

[61] Schödel J., Mole D.R., Ratcliffe P.J. (2013). Pan-genomic binding of hypoxia-inducible transcription factors. Biol. Chem..

[62] Xia X., Kung A.L. (2009). Preferential binding of HIF-1 to transcriptionally active loci determines cell-type specific response to hypoxia. Genome Biol..

[63] Sokkar P., Sathis V., Ramachandran M. (2011). Computational modeling on the recognition of the HRE motif by HIF-1: Molecular docking and molecular dynamics studies. J. Mol. Model..

[64] Bracken C.P., Whitelaw M.L., Peet D.J. (2003). The hypoxia-inducible factors: Key transcriptional regulators of hypoxic responses. Cell Mol. Life Sci..

[65] Lisy K., Peet D. (2008). Turn me on: Regulating HIF transcriptional activity. Cell Death Differ..

[66] Dal Monte M., Filippi L., Bagnoli P. (2013). Beta3-adrenergic receptors modulate vascular endothelial growth factor release in response to hypoxia through the nitric oxide pathway in mouse retinal explants. Naunyn-Schmiedeberg Arch. Pharmacol..

[67] Monte M.D., Cammalleri M., Mattei E., Filippi L., Bagnoli P. (2014). Protective Effects of 1/2 Adrenergic Receptor Deletion in a Model of Oxygen-Induced Retinopathy. Investig. Opthalmol. Vis. Sci..

[68] Hoffmann C., Leitz M.R., Oberdorf-Maass S., Lohse M.J., Klotz K.-N. (2004). Comparative pharmacology of human β-adrenergic receptor subtypes—characterization of stably transfected receptors in CHO cells. Naunyn-Schmiedebergs Arch. Pharmakol..

[69] Cernecka H., Sand C., Michel M.C. (2014). The Odd Sibling: Features of β3-Adrenoceptor Pharmacology. Mol. Pharmacol..

[70] Vij M., Drake M.J. (2015). Clinical use of the β3 adrenoceptor agonist mirabegron in patients with overactive bladder syndrome. Ther. Adv. Urol..

[71] Gauthier C., Langin D., Balligand J.-L. (2000). Beta3-Adrenoceptors in the cardiovascular system. Trends Pharmacol. Sci..

[72] Gericke A., Böhmer T., Michel M.C. (2013). β3-Adrenoceptors: A drug target in ophthalmology?. Naunyn-Schmiedebergs Arch. Pharmakol..

[73] Steinle J., Booz G.W., Meininger C., Day J.N.E., Granger H.J. (2003). Beta3-Adrenergic Receptors Regulate Retinal Endothelial Cell Migration and Proliferation. J. Biol. Chem..

[74] Steinle J.J., Zamora D.O., Rosenbaum J.T., Granger H.J. (2005). Beta3-Adrenergic receptors mediate choroidal endothelial cell invasion, proliferation, and cell elongation. Exp. Eye Res..

[75] Topcuoglu M., Aslan F. (2021). Evaluation of the Effect of a Novel Beta3-Adrenergic Agonist on Choroidal Vascularity. Investig. Opthalmol. Vis. Sci..

