# Supplementary material for: HIF-1-Dependent Induction of β3 Adrenoceptor: Evidence from the Mouse Retina
Source: Cells. 2022 Apr 8;11(8):1271. doi: 10.3390/cells11081271 (PMC9029465; doi:10.3390/cells11081271)
Supplement: Supplementary file 1 [file cells-11-01271-s001.zip › cells-1671391-supplementary.pdf]

HIF-1-dependent induction of β3 adrenoceptor: evidence from the mouse retina

Rosario Amato[1,*], Francesco Pisani[2,*], Emiliano Laudadio[3,*], Maurizio Cammalleri[1], Martina Lucchesi[1], Silvia Marracci[1], Luca Filippi[4], Roberta Galeazzi[5], Maria Svelto[2,6,7], Massimo Dal Monte[1,§,$] and Paola Bagnoli[1,§]

1 Department of Biology, University of Pisa, Pisa, Italy; rosario.amato@biologia.unipi.it (R.A.); maurizio.cammalleri@unipi.it (M.C.); martina.lucchesi@student.unisi.it (M.L.); silvia.marracci@unipi.it (S.M.); massimo.dalmonte@unipi.it (M.D.M.); paola.bagnoli@unipi.it (P.B.)

2 Department of Biosciences, Biotechnologies and Biopharmaceutics, University of Bari Aldo Moro, Bari, Italy; francesco.pisani@uniba.it (F.P.)

3 Department of Materials, Environmental Sciences and Urban Planning, Polytechnic University of Marche, Ancona, Italy; e.laudadio@staff.univpm.it (E.L.)

4 Department of Clinical and Experimental Medicine, Division of Neonatology and NICU, University of Pisa, Pisa, Italy; luca.filippi@unipi.it (L.F.)

5 Department of Life and Environmental Sciences, Polytechnic University of Marche, Ancona, Italy; r.galeazzi@staff.univpm.it (R.G.)

6 Institute of Biomembranes and Bioenergetics, National Research Council, Bari, Italy; maria.svelto@uniba.it (M.S.)

7 National Institute of Biostructures and Biosystems (INBB), Rome, Italy; maria.svelto@uniba.it (M.S.)

* Correspondence: contributing authors

§ Equal contributing senior authors

$ Correspondence: massimo.dalmonte@unipi.it (M.D.M.); Tel.: +39-050-2211426

Supplementary information

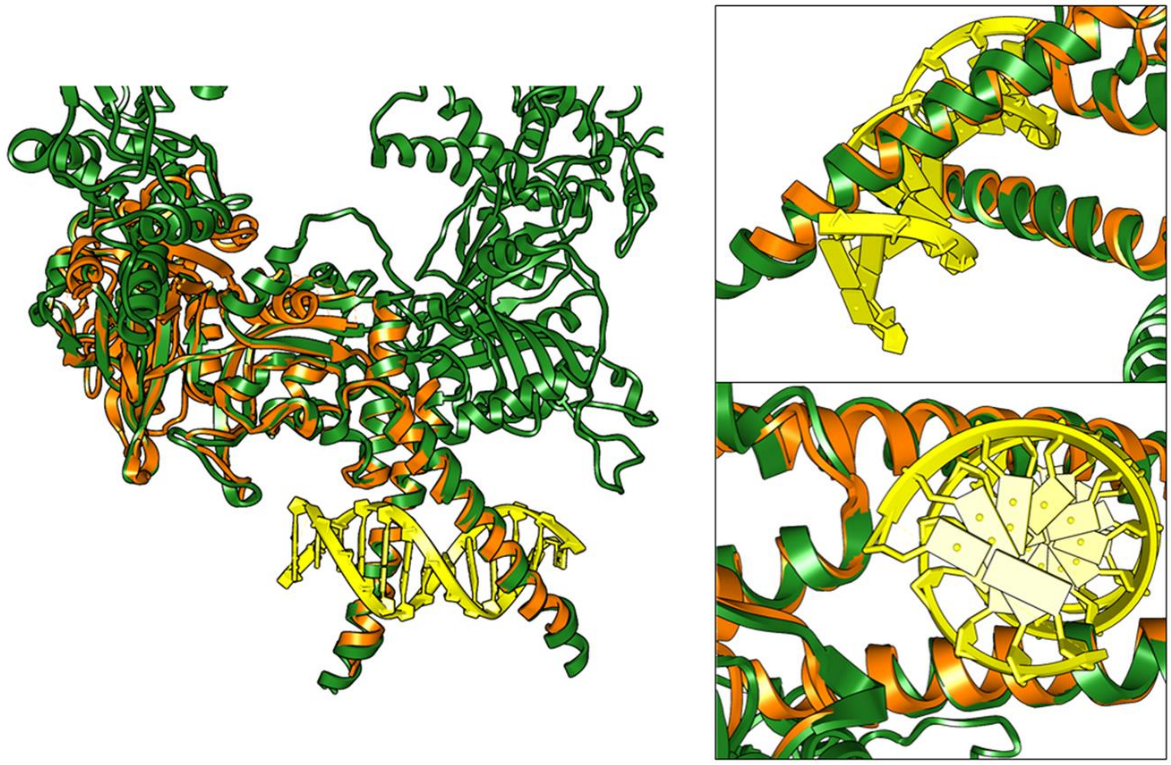

Figure S1. Close superimposition of the heterodimeric interface from 4zpr (orange) with the HIF1α homodimer model (green): full structure and focus on DNA binding domain (boxes).
